# Occlusal characteristics and oral health-related quality of life in adults operated due to sagittal synostosis in childhood: a case–control study with 26 years of follow-up

**DOI:** 10.1007/s00381-023-05871-x

**Published:** 2023-02-08

**Authors:** Johanna Julku, Niina Salokorpi, Tuula Savolainen, Ville Vuollo, Pertti Pirttiniemi, Anna-Sofia Silvola

**Affiliations:** 1 Department of Oral and Maxillofacial Diseases, Oulu University Hospital, Oulu, Finland; 2 Medical Research Center Oulu, Oulu University Hospital and University of Oulu, Oulu, Finland; 3 Department of Neurosurgery, Oulu University Hospital, OYS, Oulu, Finland; 4 Research Unit of Clinical Neuroscience, Oulu University Hospital and University of Oulu, Oulu, Finland; 5 Research Unit of Population Health, Faculty of Medicine, University of Oulu, Oulu, Finland

**Keywords:** Scaphocephaly, Malocclusion, Orthodontic treatment, Satisfaction

## Abstract

**Purpose:**

The aim of this case–control study was to investigate occlusal characteristics, received orthodontic treatment, oral health-related quality of life (OHRQoL), and satisfaction with dental esthetics in adults operated due to sagittal synostosis.

**Methods:**

The study group consisted of 40 adults (25 males, 15 females, mean age 27.4 years, range 18–41) who were operated due to isolated sagittal synostosis in childhood. The control group comprised 40 age and gender-matched adults. Occlusal characteristics were evaluated clinically during study visits. Information on the previous orthodontic treatment was collected from dental records. OHRQoL was measured using the 14-item Oral Health Impact Profile (OHIP-14), and satisfaction with dental esthetics was evaluated using a visual analogue scale.

**Results:**

No statistically significant differences were found between the patient group and the controls in malocclusion traits (overjet, overbite, molar relationships, crossbite, scissor bite), previous orthodontic treatment, pre-treatment malocclusion diagnoses, OHIP variables, or satisfaction with dental esthetics. However, there was a tendency toward increased overjet and overbite in scaphocephalic patients.

**Conclusion:**

It seems that adults with scaphocephaly operated in childhood do not differ from the average population in terms of occlusion, received orthodontic treatment, or oral health-related well-being.

## Introduction

Premature closure of one or more cranial sutures, craniosynostosis, is a relatively common birth anomaly affecting from 4.3 to 7.2 in 10,000 live births [1–3]. In the majority of the cases, there is an isolated, non-syndromic condition [4, 5]. The incidence of craniosynostosis has shown an increase in recent years, mostly seen in the cases with non-syndromic single-suture craniosynostosis [2, 3].

The fusion of the sagittal suture is the most common type of non-syndromic, unisutural craniosynostosis comprising 45 to 68% of the cases [2, 6, 7]. Males have been shown to be more affected than females, with a ratio from 2.2:1 to 3.9:1 [2, 6, 7]. The premature fusion of the sagittal suture causes typical scaphocephalic head shape by restricting and narrowing the growth of the skull in the bilateral direction with compensatory elongation in the anteroposterior direction. In addition, bifrontal or occipital bossing is seen [8].

It has been shown that in sagittal synostosis also, the cranial base has the same scaphocephalic shape as the calvarial part of the skull, although less severe [9, 10]. Most of the changes in the cranial base occur in the anteroposterior direction, especially in the posterior cranial base [11]. This elongation in the anteroposterior direction causes overrotation of the cranium and a reduction in the cranial base angle in scaphocephalic patients [12]. Early cranial vault surgery is performed during the first year of life to ensure the normalization of the growth of the skull and to provide sufficient space for the growing brain. It has been shown that the surgical repair of the cranium also has an impact on postsurgical remodeling of the cranial base [12, 13].

The growth of the cranial base may affect the craniofacial development. Changes on anterior and posterior cranial bases have an influence on the skeletal relationship of the maxilla and the mandible [14, 15]. An association of longer anterior cranial base and wider cranial base angle with the development of Class II malocclusion has been stated [14]. However, the role of the cranial base angle for the development of Class II malocclusion has been questioned [16]. A few studies looking at the occlusal and skeletal characteristics of operated and unoperated scaphocephalic patients have shown the wide cranial base angle, increased value for overjet, and the higher percentage of Class II malocclusion [17, 18].

Malocclusions have been found to be associated with lower oral health-related quality of life (OHRQoL), which includes physical, psychological, and social impacts of oral health [19]. OHRQoL is an essential part of health and well-being and is recognized by the World Health Organization (WHO) as part of an oral health program [20]. The Oral Health Impact Profile (OHIP-14) is a widely used 14-item questionnaire designed to measure the patients’ own perception of the impact of oral health conditions on the OHRQoL [21]. A recent study reported that patients operated due to non-syndromic craniosynostoses did not have reduced health-related quality of life (HRQoL) [22]. Adult non-syndromic craniosynostosis patients have been found to be generally satisfied with their facial appearance [23].

Although non-syndromic craniosynostoses are relatively common, there is a lack of studies of occlusal characteristics in these patients. In addition, OHRQoL, satisfaction with dental esthetics, and facial pain have not been previously assessed in patients with non-syndromic craniosynostosis.

The aim of this case–control study was to evaluate occlusal characteristics in adult patients previously operated due to sagittal synostosis and to investigate their history of orthodontic treatment. The further aims were to evaluate patients’ OHRQoL and their satisfaction with dental esthetics in comparison with their peers.

## Methods

The patient cohort of this study comprised all patients with craniosynostoses who were treated in the Oulu University Hospital since 1977. Patients who were 18 years or older by December 2015 and had isolated non-syndromic craniosynostosis were invited to participate in the study. A total of 61 patients agreed to participate. Patients with metopic, coronal, lambdoid, multiple suture synostosis, or hydrocephalus were excluded from the study (*n* = 21). Data on long-term follow-up of the patients treated due to sagittal suture synostosis and a description of the study protocol have been published earlier [23, 24].

The final study group consists of 40 patients (25 males, 15 females, mean age 27.4 years, range 18–41) who had been surgically treated for sagittal synostoses. The study patients were operated due to sagittal synostoses between 9 days and 45 months (mean age 5.7 months) of age. Operative treatment included linear parasagittal craniotomies with silicone membrane 105 interposition (*n* = 9), suturectomy together with dural split (*n* = 4), suturectomy without dural split 106 (*n* = 3), and various forms of H-plasty with or without barrel stave osteotomies of the temporal 107 bone (*n* = 24). The coronal and lambdoid sutures were kept intact in all cases. The age and gender-matched controls (*n* = 40) were randomly selected from the Finnish Population Register Centre [23].

The study was performed in accordance with the Declaration of Helsinki on Ethical Principles for Medical Research. The study was approved by the Ethics Review Committee of the Northern Ostrobothnia Hospital District (No. 86/2013). Patients and controls gave an informed consent for the study.

### Clinical examination

The clinical examination included registration of occlusion that was performed by one of the authors who was a resident in orthodontics (T.S.). Overjet and overbite were measured from the most labial central incisor in maximum intercuspal position using a manual scaler. The open bite was registered from both posterior and anterior areas. Lateral crossbite and scissor bite were registered according to the registration by Björk et al. [25]. Molar relationships were registered using Angle’s classification and divided into three categories: Class I, Class II and Class III. Bilateral half-cusp-Class II was categorized as “‘Class II.” In cases with asymmetric molar relationships, unilateral Class II was categorized as “‘Class II” and unilateral Class III as “‘Class III.”

### Previous orthodontic treatment

The participants were interviewed about their previous orthodontic treatment in connection with the examination. The orthodontic treatment history was dichotomized into two categories: (1) “orthodontic treatment history” and (2) “no previous orthodontics treatment.” With the permission of the participants, the orthodontic diagnosis and the previous orthodontic data of those who had undergone orthodontic treatment were collected from dental clinics where the treatment was performed. The first author, a specialist in orthodontics (J.J.), reviewed all the dental records available and categorized the data based on the main pre-treatment diagnoses and orthodontic appliances used.

### OHRQoL

A standardized self-completed questionnaire was filled in by all participants prior to the clinical examination. The questionnaire included questions assessing OHRQoL. The 14-item Oral Health Impact Profile (OHIP-14) was used to measure OHRQoL. The OHIP has been developed by Slade and Spencer [26]. The Finnish version has been found to be valid and reliable [27, 28]. OHIP-14 includes seven conceptual dimensions: functional limitation, physical pain, psychological discomfort, physical disability, psychological disability, social disability, and handicap. The frequency of each impact during the preceding month was asked on a 5-point scale. Responses were coded as follows: 0 = “never,” 1 = “hardly ever,” 2 = “occasionally,” 3 = “fairly often,” and 4 = “very often.” The OHIP outcomes consisted of the OHIP severity score, dimensions, and the prevalence of OHIP items reported occasionally, fairly often, or very often (OFoVO). The OHIP-14 severity score (potential range 0–56) was calculated by summing ordinal values for 14 items, with higher scores indicating lower OHRQoL. The total score for each dimension (potential range 0 − 8) was calculated by summing the scores of two questions.

### Satisfaction with dental esthetics

Self-rated satisfaction with dental esthetics was measured by using the 100-mm Visual Analogue Scale (VAS) with the question: “How satisfied are you with your current dental appearance?” A response of 0 mm meant “very unsatisfied,” and a response of 100 mm meant “very satisfied.”

### Statistical analysis

Statistical analyses were implemented by R software environment version 4.1.0. The Chi-square test and Mann–Whitney *U* test were used for the comparison of the patient and the control group. *P* values below 0.05 were considered significant.

## Results

The prevalence of malocclusion traits in the patient and control groups is presented in Table 1. The most common malocclusions in both groups were Class II malocclusion and increased overbite. There was a tendency toward a higher prevalence of increased overjet and increased overbite in the patient group (overjet ≥ 4 mm in 37.5% of patients vs. 25% of controls, overbite ≥ 5 mm in 30% of patients vs. 15% of controls), but the differences between the groups were not statistically significant.

**Table 1:** Prevalence of malocclusion traits in the patient and control groups

	Patients (*n* = 40)		Controls (*n* = 40)		*P*
	*n*	%	*n*	%	
Overjet (mm)					
1 − 3	25	62.5	30	75.0	N.S.
4 − 5	12	30.0	9	22.5	
≥ 6	3	7.5	1	2.5	
Overbite (mm)					
≤ 0	2	5.0	2	5.0	N.S.
1 − 4	26	65.0	32	80.0	
≥ 5	12	30.0	6	15.0	
Molar relationships					
Class I	28	71.8	31	77.5	N.S.
Class II	10	25.6	7	17.5	N.S.
Class III	1	2.6	2	5.0	N.S.
Posterior crossbite	5	12.5	6	15.0	N.S.
Scissor bite	4	10.0	1	2.5	N.S.

*N.S*. no significant

In the interviews, 23 patients and 24 controls reported that they had undergone orthodontic treatment. Two of the patients had undergone orthognathic surgery (bisagittal split osteotomy (BSSO)). The distribution of main diagnoses before orthodontic treatment did not differ significantly between the groups (Table 2). Functional and fixed appliances were the most commonly used ones in both groups (Table 3). There were no statistically significant differences in the prevalence of the appliances used.

**Table 2:** Main pre-treatment diagnoses in the patients and controls who had received orthodontic treatment

	Patients (*n* = 23)		Controls (*n* = 24)		*P*
	*n*	%	*n*	%	
Class II	10	43.5	8	33.3	N.S.
Class III	1	4.3	1	4.2	N.S.
Deep bite	2	8.7	3	12.5	N.S.
Anterior crossbite	1	4.3	1	4.2	N.S.
Posterior crossbite	1	4.3	2	8.3	N.S.
Crowding	6	26.1	5	20.8	N.S.
Hypodontia	0	0.0	2	8.3	N.S.
Missing information	2	8.7	2	8.3	N.S.

*N.S*. no significant

**Table 3:** Prevalence of previous orthodontic treatment and used appliances in the patient and control groups

	Patients (*n* = 40)		Controls (*n* = 40)		*P*
	*n*	%	*n*	%	
Previous orthodontic treatment	23	57.7	24	60.0	N.S.
Interceptive treatment	3	7.5	5	12.5	N.S.
Maxillary expansive device	2	5.0	3	7.5	N.S.
Functional appliance	15	37.5	15	37.5	N.S.
Face mask or chin-cup	0	0.0	1	2.5	N.S.
Extraction of permanent teeth	3	7.5	2	5.0	N.S.
Fixed appliance	10	25.0	12	30.0	N.S.
Orthognathic surgery	2	5.0	0	0.0	N.S.

Interceptive treatment: extraction of deciduous teeth, lingual arch, palatal arch. Maxillary expansive device: QH, RME. Functional appliance: headgear, eruption guidance appliance, activator. Orthognathic surgery: bilateral sagittal split osteotomy (BSSO)

*N.S*. no significant

OHIP-14 mean score was 3.6 (SD 6.2) in patients and 3.2 (SD 3.5) in controls. This difference between the groups was statistically insignificant. No statistically significant differences between the groups were found in any of the OHIP dimensions or OHIP prevalence (items reported “occasionally,” “fairly often,” or “very often”). Satisfaction with dental esthetics did not differ significantly between patients and controls, with satisfaction on the VAS scale being 7.0 (SD 2.2) and 7.0 (SD 1.8), respectively.

## Discussion

The present case–control study aimed to evaluate dental characteristics and received orthodontic treatment in adults operated due to sagittal synostosis in childhood. The results of the study did not show any significant differences in occlusal traits, previous orthodontic treatment, OHRQoL, or satisfaction with dental esthetics in the sagittal synostosis patients compared to the controls.

The prevalence of malocclusion traits in the patient and control groups in this study was in line with the normal adult population [29, 30]. In the patient group, there was a tendency toward increased overjet and increased overbite, which are typical occlusal features in Finnish adults, being associated with Class II malocclusion [30]. Due to the small sample size, the differences between the groups did not reveal statistical significance. The distribution of the molar relationships in both groups followed closely those reported previously in the Finnish population (Class I 68.2%, Class II 24.2%, and Class III 3.1%) [31].

The tendency toward increased overjet is in accordance with the previous findings of Lebuis et al. [18] who found that children with scaphocephaly had a clinically increased prevalence of Class II malocclusion compared to controls. In turn, the tendency toward deep bite in the present study is not supported by previous studies [17, 18] and may be caused by individual variability. In the present study, scaphocephaly patients did not significantly differ from controls in terms of present or treated crossbite or scissor bite. This is also in line with Lebuis et al. [18] who reported that the maxillary width of scaphocephaly patients remained within normal limits.

As the majority of the participants in this study had received orthodontic treatment, pre-treatment diagnoses and the orthodontic appliances used were defined from previous dental records. The most orthodontic appliances used in both groups were functional (including headgear, eruption guidance, and activators) and fixed ones, reflecting the common orthodontic practice in Finland [32]. The prevalence of both patients and controls who underwent orthodontic treatment was relatively high in relation to a previous population-based study [29]. The high prevalence of orthodontic treatment in the control group may be explained by the fact that the controls were collected from the nearby area of University Hospital, where the availability of specialized orthodontic treatment is considerably high. The patients with sagittal synostoses did not differ from their controls in terms of their previous orthodontic diagnosis and orthodontic treatment history. However, two cases in the patient group had undergone orthognathic surgery to correct mandibular retrognathia.

The growth changes of the cranial base are greatest during the first 2 to 3 years of life. The anterior cranial base grows more and matures earlier than the posterior cranial base [33, 34]. Surgical correction for sagittal synostosis is preferably performed before 1 year of age. A study looking at the differences in the growth of the cranial base between unoperated and operated sagittal synostosis patients showed that there is a reduction seen in the cranial base angle in unoperated patients, whereas the angle remains stable in operated patients [12]. Also, in their study, lengthening of the anterior cranial base was seen after surgery. When looking at the morphology of the cranial base, it was found that the angle of the cranial base did not significantly differ between different facial patterns [34, 35]. The anterior cranial base was found to be slightly longer for the Class II group, but the posterior cranial base was significantly shorter for the Class III group [35]. Based on the consistent findings of the patients and controls in the present study, it can be assumed that operated sagittal synostosis does not play a major role in the formation of different occlusal features.

Based on the results of the present study, operated sagittal synostosis is not associated with lower oral health-related physical, psychological, or social well-being. This is in line with previous results of Shavlokhova et al. [22] who reported that patients with operated non-syndromic craniosynostoses did not show any general quality of life limitations in their lives compared to the average population.

The strength of this study was its case–control design and 26.5 years of mean follow-up [23]. The clinical examination was performed by one experienced dentist to avoid inter-examiner error. OHRQoL was measured with the OHIP-14 questionnaire, which is the most widely used instrument to evaluate OHRQoL and has been tested to be reliable and valid [21, 28, 36]. A limitation of the study was the cross-sectional study design as part of the participants had received orthodontic treatment; therefore, data of pre-treatment malocclusion in those with previous orthodontic treatment was based on patient records.

Although this study population also included adults who had received orthodontic treatment, it can be suggested that the possible influence of operated sagittal synostosis on occlusion is at most minor or moderate, and individual deviations of occlusal characteristics are more prominent. In the future, longitudinal case–control studies during growth would give more information on craniofacial growth and occlusion in scaphocephaly patients.

In conclusion, the adults operated due to sagittal synostoses do not differ from the average population in terms of occlusion, received orthodontic treatment, oral health-related well-being, or satisfaction with dental esthetics. However, there may be a tendency toward increased overjet and overbite, which emphasizes the importance of screening for orthodontic treatment need during the growth of sagittal synostoses patients.

## Data Availability

Raw data are not provided. All data are summarized in the provided tables.
